# Routine health information system utilization for evidence-based decision making in Amhara national regional state, northwest Ethiopia: a multi-level analysis

**DOI:** 10.1186/s12911-021-01400-5

**Published:** 2021-01-26

**Authors:** Moges Asressie Chanyalew, Mezgebu Yitayal, Asmamaw Atnafu, Binyam Tilahun

**Affiliations:** 1 Department of Health Informatics, Institute of Public Health, College of Medicine and Health Sciences, University of Gondar, P.O. Box: 196, Gondar, Ethiopia; 2 Amhara National Regional Sate Health Bureau, Bahir Dar, Ethiopia; 3 Department of Health Systems and Policy, Institute of Public Health, College of Medicine and Health Sciences, University of Gondar, Gondar, Ethiopia

**Keywords:** Routine health information system (RHIS), Health management information system (HMIS), Evidence-base decision (EBD), Health facilities, Department heads

## Abstract

**Background:**

Health Information System is the key to making evidence-based decisions. Ethiopia has been implementing the Health Management Information System (HMIS) since 2008 to collect routine health data and revised it in 2017. However, the evidence is meager on the use of routine health information for decision making among department heads in the health facilities. The study aimed to assess the proportion of routine health information systems utilization for evidence-based decisions and factors associated with it.

**Method:**

A cross-sectional study was carried out among 386 department heads from 83 health facilities in ten selected districts in the Amhara region Northwest of Ethiopia from April to May 2019. The single population proportion formula was applied to estimate the sample size taking into account the proportion of data use 0.69, margin of error 0.05, and the critical value 1.96 at the 95% CI. The final sample size was estimated at 394 by considering 1.5 as a design effect and 5% non-response. The study participants were selected using a simple random sampling technique. Descriptive statistics mean and percentage were calculated. The study employed a generalized linear mixed-effect model. Adjusted Odds Ratio (AOR) and the 95% CI were calculated. Variables with *p *value < 0.05 were considered as predictors of routine health information system use.

**Result:**

Proportion of information use among department heads for decision making was estimated at 46%. Displaying demographic (AOR = 12.42, 95% CI [5.52, 27.98]) and performance (AOR = 1.68; 95% CI [1.33, 2.11]) data for monitoring, and providing feedback to HMIS unit (AOR = 2.29; 95% CI [1.05, 5.00]) were individual (level-1) predictors. Maintaining performance monitoring team minute (AOR = 3.53; 95% CI [1.61, 7.75]), receiving senior management directives (AOR = 3.56; 95% CI [1.76, 7.19]), supervision (AOR = 2.84; 95% CI [1.33, 6.07]), using HMIS data for target setting (AOR = 3.43; 95% CI [1.66, 7.09]), and work location (AOR = 0.16; 95% CI [0.07, 0.39]) were organizational (level-2) explanatory variables.

**Conclusion:**

The proportion of routine health information utilization for decision making was low. Displaying demographic and performance data, providing feedback to HMIS unit, maintaining performance monitoring team minute, conducting supervision, using HMIS data for target setting, and work location were factors associated with the use of routine health information for decision making. Therefore, strengthening the capacity of department heads on data displaying, supervision, feedback mechanisms, and engagement of senior management are highly recommended.

## Background

Health Information System (HIS) is the basis of the health system and the key to making evidence-based health policy decisions [1]. It is the intersection of healthcare’s business processes and information systems to deliver better healthcare services [2, 3]. Routine health information system (RHIS) is a part of HIS, that generates data at regular intervals (no longer than a year) that have been collected at public and private health facilities and institutions, as well as at community-level health posts and clinics [4]. Ethiopia has implemented the Health Management Information System (HMIS) since 2008 to collect routine data as a primary source of information in public and private facilities [5].

The HMIS was one of the seven components of the Health Sector Development Program-III (HSDPIII) and is one of the four transformation agendas (Information Revolution) that the country has formulated in its Health Sector Transformation Plan (HSTP), 2015–2019. The country developed Information Revolution Roadmap (IRR) in 2017 with the foremost aim of enhancing the culture of routine health information use for evidence-based decisions at the lower level. As a result, a myriad of efforts has exerted to realize the objectives of IRR across the country. Revising key performance monitoring indicators, standardizing data recording and reporting tools, conducting capacity building training, implementing electronic Medical records (eMR), introducing District Health Information System (DHIS_2_), and installing SMART care at health facility levels were some of the efforts exerted among others [5–7].

Public health decision-making is critically dependent on the timely availability of sound data [8]. Shreds of evidence showed that the proportion of information-use for evidence-based decisions was demonstrated only in 52% [9] and 53% [10] of the health facilities in Mexico and South Africa, respectively. Likewise, a single study done in India and Tanzania indicated that department heads experienced missed opportunities for using data for decision making [11]. Empirical evidence in Ethiopia showed that the proportion of information use for decision making was inconsistent from place to place across the country. The highest was reported in North Gondar (78.5%) [12] and Hadya (69.3%) [13], and the lowest was in Jimma (32.9%) [14]. However, the study done in Ayder referral hospital at Mekele revealed that no evidence was found regarding the use of information generated by the facility for decision making [15]. Shreds of evidence from East Gojam Zone of Amhara region indicated that the proportion of routine health information use for evidence-based decision making among health professionals was 45.8% [16].

Routine health information system (RHIS) use for decision making was affected by multiple factors. An assessment conducted in Tanzania by Measure Evaluation revealed that lack of analytic and data use skills were constraints of information use [11]. Teklegiorgis et al. reported that a friendly format for reporting and managers provide regular feedback to their staff were found to be significantly associated with health information utilization[17]. Likewise, Adane et al. and Asemahagn et al. mentioned that provisions of technical supports, presence of computers, the practice of conversion of data into information, residence, data management knowledge, workload, and computer skill enhanced the utilization of HMIS for evidence-based decisions making [18, 19].

Departments in health facilities are primary sources of routine health information in the healthcare system of the country. Hence, the country has made immense efforts through the information revolution agenda since 2017 to enhance the utilization of routine health information for evidence-based decisions at lower levels of the healthcare system. The findings of previous studies indicated that the use of routine health information for decision making varied from place to place but failed to account for individual and organizational level variations that affect the enhanced use of routine health information [12, 13, 16–20]. Failing to capture the variations at different level did not provide appropriate parameters estimates [21, 22]. Hence, the use of routine health information for decision making in the study area remains a problem. Therefore, this study was conducted to assess the proportion of routine health information system utilization and its determinants among department heads in the Amhara Region public health facilities. Hence, taking in to account the limitation of previous studies, this study employed a facility level cross-sectional study with multilevel analysis.

## Methods

### Study design and settings

The study employed a cross-sectional design and was conducted in ten selected districts of Amhara National Regional State in the Northwest of Ethiopia from April to May 2019. The region was subdivided administratively into 12 zones and three city administrations, and 183 districts. According to the 2020 fiscal year, the total population of the region was estimated to be 22,191,890 (11,317,864 males and 10,874,026 females); and 3560 Health Posts, 858 Health Centers, and 81 Hospitals were providing healthcare services to the community [23].

### Study population, sample size and sampling procedures

The study population was all department heads working at health facilities in the Amhara region. Information use is a composite indicator that cannot be measured by a single indicator at a point in time. It is the process that encompasses problem identification, prioritization, action plan development, implementation, and following up, and providing regular feedback that requires a minimum of three months stay. Hence, the study recruited the heads of departments who were working at least for three months. Moreover, the health facilities require at least six months functioning the HIS activities effectively. As a result, the study included health facilities that provided healthcare services for more than six months. Routine health information is carried out primarily by departments that provided healthcare services. Hence, department heads that are supportive of the healthcare services such as human resources, finance, and guardian were excluded from the study. The total number of medical departments in the 83 facilities was 1218. The sample size was calculated using a single population proportion formula by considering anticipated proportion of data use 0.69 [13], a margin of error 0.05, and the square of the normal deviate at the required confidence level at 95% was 1.96. With considering a factor 1.5 as design effect and 5% non-response rate the final sample size was estimated to be 394 [24]. Hence, the total sample size was proportionally allocated to the selected health facilities [25].

The study applied a multi-stage sampling method to recruit study subjects. At the first stage, the team selected ten districts randomly. The estimated sample size (394 department heads) was distributed to all health facilities in the ten districts using the population to proportion size formula. Following that, the sampling frame was developed, which contained the list of departments in each health facility. Secondly, department heads were included in the study using simple random sampling techniques from all facilities in the selected districts.

### Data collection tool and procedure

The data collection tool was developed from the Performance of Routine Information System Management (PRISM) tool and adapted to the local context [26, 27]. The tool collected information on technical, behavioral, and organizational determinant factors of routine health information use. Availability of resources, training, supervision, finances, information distribution, and promotion of the culture of information were organizational determinants. Behavioral factors were data demand, data quality checking skills, problem-solving for HIS tasks, competence in HIS tasks, confidence levels for HIS tasks, and motivation. Besides, the complexity of the reporting form, procedures, HIS design, computer software, and IT complexity were technical determinants.

Forward translation to the Amharic language was done independently by a naïve and public health professional translators. It was also back-translated to the original English language by the investigator for consistency check. Following this, the tool was piloted in two districts (Debre Tabor and Enjibara) that had comparable settings to the study sites. Reliability analysis was carried out before starting data collection to see whether the tool can measure the true value of the outcome variable. The overall level of reliability index (Cronbach's alpha value) was 0.89. The result was above 0.7 (acceptable level) that indicated the tool was reproducible to the local settings [28].

### Operational definitions and study variables

The outcome variable was the proportion of routine health information utilization, measured using five core indicators identified from the PRISM tool. The presence of feedback provided by department heads to health workers in the department, evidence on the use of information for decision making, key performance indicators, evidence on health coverage, and target achievements against the plan were the indicators applied to determine the level of routine health information-use for decision making. The mean value for the five indicators was calculated to categorize the level of information use among study participants. Study participants who scored above mean value was considered as “have good information use” or else if they scored equal and below the mean value “have poor information use” for evidence-based decision making.

Organizational determinants include governance, planning, supervision, information distribution, availability of resources for HIS, training, and promotion of a culture of information use. Besides, the technical determinants encompass the complexity of the reporting form, procedures, HIS design, computer software, and IT complexity. Likewise, the behavioral determinants incorporate data quality checking skills, problem-solving for HIS tasks, competence in HIS activities, and motivation.

### Statistical analysis

Data were entered in to EpiData version 3.1 and exported in a csv format to R software version 3.4.3 for further analysis. Descriptive statistics mean and percentage were calculated. A bi-variate analysis was conducted to identify potential candidate variables for multivariable analysis. Predictor variables that were significant at *p *value  < 0.2 were entered into the multivariable analysis. Independent multivariable analysis was fitted for level one and level two variables. The effect of multicollinearity was examined using the score of Variance Inflation Factors (VIF), and Variables with VIF score greater than 10 were excluded from the model. Forward stepwise techniques were applied to identify explanatory variables that have a significant association with the outcome variables to build the Generalized Linear Mixed Effect Model (GLMM). Finally, variables with *p *value  < 0.05 in multivariable analysis were fitted to the GLMM to quantify the effects of level one (individual-level characteristics) and level two (organizational level characteristics) variables on the observed level of information use.

The conditional model was fitted at the first step in the GLMM. Pieces of information about the log-likelihood ratio score, Intra-class correlation (ICC) score, the random variance, Achachie's Information Criteria (AIC), and Bayesian Information Criteria (BIC) were calculated. The model with non-zero log-likelihood ratio and ICC score of > 0.05 indicated the presence of cluster-level correlation (the between facility-level variance) that insinuated to fit GLMM [29].

At the second stage, random and fixed effects (intercepts and slopes) were calculated for the three consecutive models. The AIC, BIC, and tau squared scores were examined to check the adequacy of models. Embedded models with small values were considered a better model. Furthermore, Analysis of Variance (ANOVA) was fitted at each step to test the significance of additional terms (random and fixed effects) in the embedded models. A significant value for the test statistics (p < 0.05) indicated that including additional terms in the final model enabled to produce the right parameter estimate [3, 30, 31]. The Adjusted Odds Ratio (AOR), 95% CI, and *p *value were calculated for all variables in the final model; and those variables with *p *value  < 0.05 were considered as predictors of routine health information use. Furthermore, the final or global model was examined for overdispersion using the ratio of final model deviance or Pearson’s chi-square to the residual degree of freedom. Models with the ratio value close to one were considered that non-dispersed model [32]. The presence of overdispersion in a model suggests it is a bad fit [33].

## Result

### Socio-demographic characteristics of study participants

The study enrolled a total of 386 (98%) department heads in 83 health facilities. The data showed that among the 83 facilities assessed, 76 (92%) were health centers, 5(6%) were primary hospitals, and (2%) were referral hospitals.

The mean age of respondents was 28.2 years, with a standard deviation of 5.4 years. Of the total 386 study participants included, 238(62%) of them were female participants, 294(82.4%) of them were below the age of 30 years, 225(58.3%) of them were diploma graduates, and 115(29.8%) of them were clinical nurses by profession. While considering the year of experience, 239(63.7%) of the study participants had less than five years of experience, 281(74.3%) of them earned five thousand and less Ethiopian birr per month, and 233(60.4%) of them were rural dwellers. Of the total 386 department heads, 97(25.1%) of them were from Maternal and Child Health (MCH) departments, followed by Out-patient departments 76(19.7%) (Table1).

**Table 1:** Socio-demographic characteristics of study participants in health facilities in the Amhara region, Northwest Ethiopia, 2019

Variables	Number	Percent (%)
Sex (N − = 384)		
Male	146	38
Female	238	62
Age in year (N = 357)		
≤ 30	294	82.4
> 30	63	17.6
Level of education (N = 386)		
Grade ten completed	2	0.5
Diploma	225	58.3
Bachelor	155	40.2
Master	4	1.0
Profession (N − = 386)		
BSC midwifery	18	4.7
BSC nurse	51	13.2
Health officers	56	14.5
Clinical nurse diploma	115	29.8
Health information technician	42	10.9
Laboratory diploma	17	4.4
Midwifery diploma	37	9.6
Pharmacy diploma	39	10.1
Others	11	2.8
Experience in year (N = 375)		
≤ 5	239	63.7
6–10	102	27.2
≥ 11	34	9.1
Salary in ETB (N = 378)		
≤ 5000	281	74.3
5001–10,000	94	24.9
> 10,000	3	0.8
Work location (N = 386)		
Urban	153	39.6
Rural	233	60.4
Departments by type (N = 386)		
Anti-retroviral therapy (ART)	17	4.4
Emergency outpatient department	18	4.8
Facility head office	24	6.2
HMIS unit	41	10.6
Laboratory	17	4.4
Maternal and child health (MCH)	97	25.1
Out-patient department (OPD)	76	19.7
Pharmacy	42	10.9
TB clinic	16	4.1
Youth friendly service	18	5.2
Others	20	3.4

### Organizational determinants

#### Staff meeting and motivation

From a total of 386 departments assessed, 324(83.9%) conducted regular staff meetings in the last three months of which, 240(74%) conducted senior staff meetings, 310(96%) carried out all-staff meetings, and 295(91%) implemented department-level meetings. Only 43.4% of department heads received feedback from seniors to improve their performance. Among 377 respondents, 173(45.9%) agreed that the facilities arranged an incentive or motivation mechanism to improve the use of information for evidence-based decision making that in turn improved the quality of healthcare.

#### Performance monitoring team discussions

**Fig. 1:**
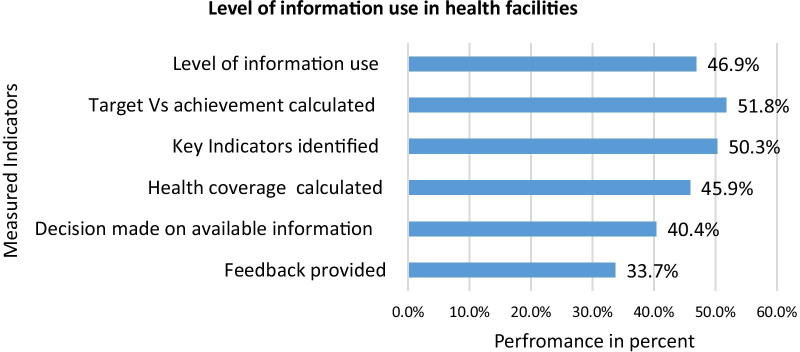
Level of information-use for evidence-based decision making in health facilities in the Amhara region, Northwest Ethiopia, 2019

Of the total 386 department heads observed, 252(65%) and 244(63%) of them mentioned Performance Monitoring Team (PMT) meetings were conducted regularly and maintained official minutes that contains the discussion points and decisions made during their meeting, respectively. Regarding PMT meeting contents, 219(89%) of minutes showed management of RHIS activities, 183(75%) indicated discussions made on RHIS findings, 163(66%) revealed the decisions made, and 124(51%) showed follow up actions taken place regarding the decisions. However, only 63(25%) showed RHIS related issues or problems referred to a district or regional level for further actions. Of the total 386 departments investigated, 200(52%) calculated target against achievement, and only 130(33.7%) provided feedback to lower-level health workers. As a result, the study revealed that only 46.9% of department heads utilized routine health information for evidence-based decisions (Fig. 1).

Among the total study participants, 245(63.6%) received annual or monthly targets on RHIS performance; however, only 189(48.9%) of them mentioned that they received senior management directives concerning the use of information, 146(37.8%) received a district office report, and 150(38.9%) had documents showing the use of information for advocacy purposes. Besides, 173(44.8%) of them participated in meetings held at the district level.

#### Display of information

Of the total 386 respondents, only 118(30.6%) and 156(40.4%) of them displayed the catchment area map and summary of demographic information such as population by age group, respectively. However, feedback provided by district office or higher organization was found only in 129(33%) of the departments (Table 2).

**Table 2:** Type of information displayed in health facilities in the Amhara region, Northwest Ethiopia, 2019

Variables	Number	Percent (%)
Catchment area map (N = 386)		
Yes	118	30.6
No	268	69.4
Demographic data (N = 386)		
Yes	156	40.4
No	230	59.6
Feedback available (N = 386)		
Yes	129	33.4
No	257	66.6
Feedback review strategy (N = 129)		
Yes	111	86.1
No	18	13.9
Feedback review personal responsibility (N = 129)		
Yes	115	89.2
No	14	10.8
Feedback indicate resource mobilization (N = 129)		
Yes	87	67.4
No	42	32.6
Feedback indicate data use for advocacy (N = 129)		
Yes	83	64.3
No	46	35.7

#### RHIS supervision

Among the total 386 department heads assessed, only 158(41%) of them received RHIS supervision from higher-level institutions. Of which, majority 148(93.7%), 146(92.4%), and 140(88.6%) explained that supervisors use a checklist, check the quality of data, and discussed performance based on target and achievement, respectively; however, only 98(62%) of supervisors sent official reports to supervisee after the supervision had taken place. Among 383 department heads, only 129(33.4%) mentioned that internal supervision was conducted regularly, and 66(51.1%) of them were provided written feedback that addressed the weaknesses and strengths of RHIS performance.

#### Planning

Among a total of 386 department heads assessed, 66(17.1%) of them had no strategic plan of the facility. However, 242(62.7%) of them had received an annual plan for the 2019 fiscal year, of which 119(49.2%) reflected the use of routine data for problem identification. Of the total 386 departments observed, the practice of data use for the target set during annual planning was observed only in 167(43.3%) of departments (Table 3).

**Table 3:** Characteristics of the planning process in selected health facilities of the Amhara region, Northwest Ethiopia, 2019

Characteristics	Frequency	Percent (%)
Strategic plan available (N − = 386)		
Yes	66	17.1
No	320	82.9
Annual plan available (N = 386)		
Yes	242	62.7
No	144	37.3
Plan reflect use of data for problem identification (N = 242)		
Yes	119	49.2
No	123	50.8
HMIS data use for target setting (N = 386)		
Yes	167	43.3
No	123	50.8

### Technical determinants

**Fig. 2:**
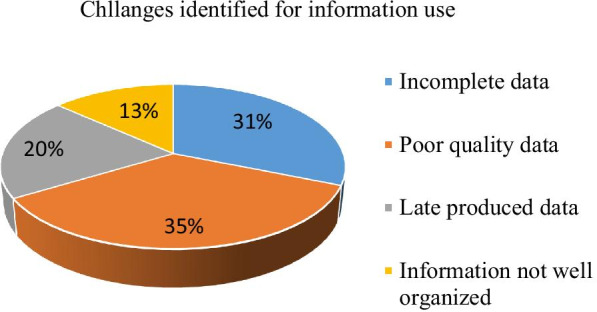
Challenges of information use for decision making in the Amhara region, Northwest Ethiopia, 2019

The study indicated that, among 384 department heads, 137(32.7%) faced challenges while using HMIS data as detailed below (Fig. 2) of which, 110(80.3%) of them provided feedback to the HMIS unit to improve the challenges, and 88(80.6%) of them explained the feedback get addressed. Among a total of 384 department heads examined, 256(66.3%) explained they had the skill to make an evidence-based decision. The study also indicated that 288(74.6%) of study participants need training on the use of data for decision making followed by data collection 152(39.4%), and data analysis 151(39.1%).

### Behavioural determinants

Among the total 380 study participants, 211(55.5%) and 119(31.3%) of them agreed that not using collected data discouraged them, and collecting information led them to feel bored, respectively. However, 342(89.8%) of respondents mentioned that collecting information was meaningful for them, 346(91.5%) explained collecting information gave them that data were needed, and 308(81.5%) of them mentioned that collection information was appreciated. Fewer 45(11.8%) of them explained that collecting information gave them forced activities.

#### Factors affecting the level of information use

The study revealed that displaying demographic and performance data, and providing feedback were individual-level predictors whereas maintaining PMT minute, issuing directives by senior management, using HMIS data for target setting, receiving HIS supervision, and location were organizational level predictors (Table 4).

**Table 4:** Factors associated with the level of information-use for decision making in the Amhara region, Northwest Ethiopia, 2019

Variables	Null model		Model with level-1 variables		Model with level-2 variables		Model with both level-1 and level-2 variables	
	AOR (95% CI)	*p *value	AOR (95% CI)	*p *value	AOR (95% CI)	*p *value	AOR (95% CI)	*p *value
Intercept	0.85 (0.59, 1.24)	0.406	0.004 (0.001, 0.02)	0.001***	0.25 (0.05, 1.15)	0.076	0.03 (0.005, 0.21)	0.001***
Display demographic data	–	–	7.46 (3.52, 15.83)	0.001 ***	–	–	12.42 (5.52, 27.98)	0.001***
Received office report	–	–	5.33 (2.58, 10.99)	0.001 ***	–	–	–	–
Supervisor provide feedback timely	–	–	1.22 (1.03, 1.45)	0.05*	–	–	–	–
Supervise staff	–	–	1.64 (1.11, 2.43)	0.05*	–	–	–	–
Staff display data for monitoring	–	–	1.48 (1.18, 1.85)	0.000 ***	–	–	1.68 (1.33, 2.11)	0.001***
Provide feedback to HMIS unit	–	–	2.93 (1.42, 6.07)	0.01**	–	–	2.29 (1.05, 5.00)	0.05*
Feedback from seniors available	–	–	2.79 (1.29, 6.07)	0.01**	–	–	–	–
PMT minute maintained	–	–	–	–	2.78 (1.29, 5.98)	0.01**	3.53 (1.61, 7.75)	0.01**
Received planned target	–	–	–	–	2.63 (1.17, 5.92)	0.05*	–	–
Senior management issued directives	–	–	–	–	3.06 (1.58, 5.90)	0.001***	3.56 (1.76, 7.19)	0.001***
Have annual plan	–	–	–	–	2.93 (1.24, 6.92)	0.05*	–	–
HMIS data use for target setting	–	–	–	–	2.20 (1.09, 4.45)	0.05*	3.43 (1.66, 7.09)	0.001***
Received HIS supervision	–	–	–	–	2.34 (1.13, 4.88)	0.05*	2.84 (1.33, 6.07)	0.01**
Location	–	–	–	–	0.27 (0.12, 0.60)	0.01**	0.16 (0.07, 0.39)	0.001***

***p value  < 0.001,**p value  < 0.01,*p value  < 0.05, ‘.’*p *value  < 0.1

The study revealed that, department heads who displayed demographic data were 12.42 (AOR = 12.42; 95% CI [5.52, 27.98]) times more likely to use the information for decision making than those who did not display demographic data. Besides, the odds of use of information for decision making was 1.68 (AOR = 1.68; 95% CI [1.33, 2.11]) and 2.29 (AOR = 2.29; 95% CI [1.05, 5.00]) times higher among department heads who displayed performance data for monitoring and provided feedback to the HMIS unit regarding data quality than their counter parts those who did not, respectively (Table 4).

The study indicated that, facilities that maintained PMT minute and received senior management directives had 3.53 (AOR = 3.53; 95% CI [1.61, 7.75]) and 3.56 (AOR = 3.56; 95% CI [1.76, 7.19]) times higher odds of the level of information-use for decisions, respectively. Besides, the level of information-use for decision making was 3.43 (AOR = 3.43; 95% CI [1.66, 7.09]) and 2.84 (AOR = 2.84; 95% CI [1.33, 6.07]) times more likely in health facilities which utilized HMIS data for target setting and received RHIS supervision, respectively. However, the level of information-use in rural facilities was 84% (AOR = 0.16; 95% CI [0.07, 0.39]) less likely than urban facilities (Table 4).

### Model appropriateness

The score of the Log-likelihood ratio for the null model in the GLMM was subtracted from the ordinary logistic regression model and resulted in -19.65784 (degree of freedom = 1). As a result, the test statistics (− 2* Log likelihood ratio) was 39.316, which indicated that the between-facility variance was non-zero. Besides, the ANOVA model employed for the null model in the GLMM and ordinary logistic regression resulted in a statistically significant chi-square test (χ^2^ = 39.316, p ≤ 0.001), which supported to include additional terms (random effect) in the model. Moreover, the ICC score in the null model was 0.35, which implied that 35% of the chance of having a good level of information use was explained by between-cluster differences (facilities) (Table 5).

**Table 5:** Summary of information for the generalized linear mixed effect model in the Amhara region, Northwest Ethiopia, 2019

Model components	Null model	Model with level-1 variables	Model with level 2 variables	Model with both level-1 and level-2 variables
Variance of random effect (τ^2^)	1.778	1.574	1.102	0.7187
LR	− 247.2	− 173.7	− 171.3	− 142.7
PCV	Reference	11.4%	37.9%	59.6%
ICC	0.351	0.324	0.251	0.179
AIC	498.3	365.4	360.6	305.5
BIC	506.2	401.0	396.1	344.9

*LR* log-likelihood ratio, *PCV* proportional change in the variance, *ICC* intra class correlation, *AIC* Akaike information criteria, *BIC* Bayesian information criteria

### Model comparison

The scores for AIC and BIC decreased from the null to the global model in a generalized linear mixed-effect model. Unlike that, the value for PCV increased in cascading models (Table 5).

### Model fit and performance diagnosis

The ratio of residual deviance to the degree of freedom for the global model (model with both individual and organizational level variables) was 0.97, which explained that the model was not over-dispersed.

### Multicollinearity test

Though a multicollinearity test was conducted for the final model, none of the variables' scores showed greater or equal to 10 for the test statistic.

## Discussion

The study indicated that the overall proportion of information-use for evidence-based decision making among department heads was very low. The finding was by far below the result reported from North Gondar and Hadiya zone, Ethiopia where the level of information-use for evidence-based decision making was 78.5% [12] and 69.3% [13], respectively. Also, it was below the study done in South Africa [34]. The possible explanation for this difference could be, this study included health facilities obtained from districts with very wide geographic coverage which were not easily accessible for support. As a result, the low level of information- use for evidence-based decision making affects the quality of healthcare services. However, the finding was higher than the study done in Jimma and Addis Ababa which reported the level of information-use as 31% [14] and 41.7% [18], respectively. The variation might be because of the difference in the study period and efforts exerted in the past two years following the development of IRR by different actors in the study area.

Among individual-level predictors, displaying demographic and performance data for monitoring and providing feedback to the HMIS unit were positively associated with the level of information-use. The higher odds of routine health information system utilization for evidence-based decision making were observed among department heads who displayed demographic and performance data for monitoring. The finding was in line with a study done in Jimma that reported displaying demographic and performance data were associated with a good level of information-use [14]. Displaying demographic data by age, sex, and location would help healthcare providers to monitor whether target groups were provided services properly according to their needs. Besides, displaying performance data based on target versus achievement supported healthcare providers to identify the area where healthcare services imbalance existed that required further improvements [35].

Likewise, the studies conducted in Dire Dawa [17], the odds of information-use for evidence-based decision making was higher among department heads who provided feedback to the HMIS unit about data quality. It is known fact that providing feedback to health departments that indicated the strength and weakness based on supervision findings would help to improve the performance of the health information system at all levels in the health system [36].

Maintaining PMT minute, issuing directives by senior management, using HMIS data for target setting, receiving HIS supervision, and location were organizational level predictors of routine health information use for evidence-based decision. The level of information use was higher among department heads those who received RHIS supervision than compared to their counterparts. Regular HIS supervision might help health workers to improve their capacity on health data analysis, interpretation, and use to make an evidence-based decision at lower levels of the health care system [16].

Similarly, high odds of information use were noted among department heads who maintained Routine Performance Monitoring Team (RPMT) minute. It was evidenced elsewhere, PMT was a core ingredient that would help to improve RHIS performance (improved data quality and enhanced use of routine information for decision making). Maintaining the discussion points and decisions made during the PMT meeting guided the overall healthcare services and activities undertaken in the health facilities. It was also mentioned in the National Information Use Guideline of Ethiopia, conducting PMT, keeping the records of PM, and translating it to the action plan as paramount activities that all health facilities were supposed to implement [37].

The odds of good information use among department heads in rural facilities were less likely than their counterparts in the urban facilities. The finding was in line with the result reported in East Gojam, Ethiopia [19], and also supported by Information Revolution Roadmap, Ethiopia [6]. This could be due to the fact that the low emphasis was given to lower-level health workers (department heads) who were engaged in data generation in rural facilities. Besides, there was limited and inadequate support provided by higher institutions (district, zone, or regional level) that helped them to improve the practice of evidence-based decision making.

In a nutshell, this study highlighted the utilization of routine health information systems among department heads for evidence-based decision making and its attributes. It uncovered the practice of feedback mechanisms, training needs, supervision modality, planning process, motivation mechanisms, and behavior of study participants on routine health information use. It may have indicated the area where further improvements are required. Besides, the finding may serve as a source of information for further studies.

The study included health facilities in selected districts that covered large geographical locations in the region. Hence, the findings of this research may have high generalizability. Moreover, it accounted for variations at different levels to delineate the effect of an individual as well as organizational level attributes using a generalized linear mixed-effect model. However, the limitations of this study were, it only indicated that the one-time study findings (since the study applied a cross-sectional design). Besides, it did not address the perception of department heads towards routine health information use for evidence-based decision making.

## Conclusion

The overall level of routine health information utilization for evidence-based decision making was low. It signified that department heads poorly utilized available information to lead program performance and managed resources for effective utilization. Individual-level factors (displaying demographic and performance data for monitoring and providing feedback to HMIS unit) and organizational level factors (maintaining performance monitoring team minute, conducting supervision, issuing directives by senior management, using HMIS data for target setting, and location) were associated with utilization of routine health information for decision making. Therefore, strengthening the capacity of department heads on data displaying for monitoring, supervision, feedback mechanisms, and engagement of senior management on RHIS activities is highly recommended. Further research is suggested in the perception of department heads towards routine health information use to enhance evidence-based practice.

## Data Availability

Data will be available up on reasonable request from the corresponding author.

## Abbreviations

AOR: Adjusted Odds Ratio
CI: Confidence Interval
DHIS: District Health Information System
EDM: Evidence-based Decision Making
HIS: Health Information System
HMIS: Health Management Information System
HSDP: Health Sector Development Plan
HSTP: Health Sector Transformation Plan
IRR: Information Revolution Roadmap
PRISM: Performance of Routine Health Information
RHIS: Routine Health Information System
